# Fertility Social Mentality Scale for Women of Childbearing Age: a scale development study

**DOI:** 10.3389/fpsyg.2026.1799068

**Published:** 2026-06-11

**Authors:** Binyang Zhang, Nanyin Bu, Shiqin Xu, Zuoshan Li

**Affiliations:** 1 Key Laboratory of Applied Psychology, Chongqing Normal University, Chongqing, China; 2 Clinical School of Medicine, Chongqing Medical and Pharmaceutical College, Chongqing, China; 3 School of Finance, Chongqing Technology and Business University, Chongqing, China; 4 School of Teacher Education, Chongqing Normal University, Chongqing, China

**Keywords:** fertility social mentality, measurement invariance, reliability, validity, women of childbearing age

## Abstract

**Objective:**

To develop a scale to measure fertility social mentality among women of childbearing age in China and to assess its psychometric properties, including reliability, validity, and measurement invariance.

**Methods:**

A total of 496, 857, 607, 632 and 362 women of childbearing age were recruited to conduct exploratory factor analysis, reliability and validity testing, and measurement invariance testing, respectively.

**Results:**

The Fertility Social Mentality Scale for Women of Childbearing Age comprises 27 items and consists of three factors: fertility social values, fertility social cognition, and fertility social emotions. Confirmatory factor analysis indicated a good fit of the three-factor structural model [*χ*^2^/*df* = 3.735, CFI = 0.918, TLI = 0.910, SRMR = 0.065, RMSEA = 0.056 (90% CI: 0.053–0.060)]. The average variance extracted (AVE) values were 0.444, 0.466, and 0.509, respectively. Discriminant validity analysis showed that the correlation coefficients among dimensions were all lower than the square roots of the AVE. Criterion analysis revealed that the total score of the scale and its individual dimensions were significantly positively correlated with fertility efficacy and fertility behavior, with correlation coefficients ranging from 0.468 to 0.727 and from 0.403 to 0.551, respectively. The Cronbach’s *α* coefficients for the total scale and the three factors were 0.924, 0.897, 0.871, and 0.892, respectively. The composite reliability (CR) values ranged from 0.765 to 0.809. The test–retest reliability coefficients for the total scale and each dimension were 0.953, 0.949, 0.924, and 0.890, respectively.

**Conclusion:**

The developed Fertility Social Mentality Scale for Women of Childbearing Age shows encouraging evidence of structural validity, internal consistency, test–retest reliability, and measurement invariance. The scale may serve as a preliminary instrument for assessing fertility social mentality among women of childbearing age in China.

## 1 Introduction

China’s total fertility rate has remained below the replacement level since 1992, marking a significant shift in population development trends. In response, fertility policies have gradually been relaxed; however, these changes have not led to a significant increase in fertility rates. The persistently low fertility trend is affecting the overall sustainable development of Chinese society (Zuo, 2023). In this context, fertility decision-making is increasingly understood as being shaped not only by structural and policy conditions, but also by proximal psychological factors such as attitudes, intentions, desires, and value-laden evaluations related to childbearing (Hashemzadeh et al., 2021). From this perspective, the concept of social mentality provides a useful framework for understanding socially embedded psychological processes. However, research on social mentality toward fertility remains at an early stage, and the field still lacks a dedicated instrument capable of assessing this broader and socially embedded fertility-related orientation in a systematic way. Existing studies have been largely theoretical, with limited quantitative work examining its internal structure and correlates. Accordingly, there is a need to develop a conceptually clearer and psychometrically grounded measure of fertility social mentality, rather than another general measure of fertility attitudes alone.

To address this need, the present study defines fertility social mentality as a socially embedded and context-sensitive configuration of fertility-related cognition, emotion, and value orientation through which women interpret, feel about, and normatively evaluate fertility issues. Importantly, this construct is not intended as a simple extension or repackaging of traditional fertility attitudes. Instead, it represents a higher-order psychological system integrating contextual interpretation, affective responses, and value-based evaluations under specific socio-cultural conditions. This construct is conceptually related to, but distinct from, other fertility-related psychological constructs. For example, fertility intention generally refers to an individual’s plan or willingness to have a child, fertility desire reflects the subjective wish for childbearing, and fertility attitude refers to a more general evaluative stance toward fertility and childbearing (Söderberg et al., 2013; Naghibi et al., 2019).

From a theoretical perspective, fertility social mentality can be conceptualized as a multi-pathway psychological system through which socially contextualized information is processed and translated into fertility-related judgments and tendencies. Within this framework, fertility-related social cognition can be understood as one important dimension of fertility social mentality, because it reflects how individuals interpret fertility-related norms, opportunities, constraints, and support within their broader social environment.

Clore and Schnall (2005) suggests that emotions have a direct impact on attitudes. Emotions are rapid evaluative responses to stimuli, and these responses directly influence decision-making tendencies. Building on this view, in the context of fertility decision-making, these emotional responses function as rapid evaluative signals that shape approach–avoidance tendencies, thereby influencing how individuals prioritize and process fertility-related information (Fazio, 2001). From this perspective, fertility-related emotions may be treated as a second dimension of fertility social mentality, because they capture the affective meanings and motivational tendencies attached to childbearing.

In addition, fertility social values refer to an individual’s psychological tendency to evaluate the importance of various aspects of fertility, directly reflecting their choices and trade-offs regarding different fertility goals, methods, and preferences (Tong and Zhang, 2002). Values are not only standards of evaluation but are also systematically related to behavioral preferences and action tendencies (Schwartz, 2012). In this sense, fertility social values serve as relatively stable evaluative standards that organize cognitive appraisals and emotional responses into coherent decision tendencies. Therefore, fertility social values can be regarded as a further dimension of fertility social mentality, insofar as they provide relatively stable evaluative orientations that guide how fertility is judged and prioritized.

Taken together, the preceding discussion suggests that fertility social mentality can be provisionally conceptualized in terms of fertility-related cognition, emotion, and value orientation. Guided by social mentality theory, the present study treated these three components as an initial framework for operationalizing the construct (Yang, 2006). This framework was not assumed to be final or exclusive; rather, it was adopted as a theoretically informed starting point to be examined empirically through scale development procedures (Clark and Watson, 1995; Lambert and Newman, 2023). Compared with existing fertility-related instruments that primarily assess intentions, desires, or general attitudes, the present study aims to develop a measure of fertility social mentality that complements these instruments by capturing a broader socio-psychological orientation toward fertility embedded in social context, policy discourse, perceived norms, and value-laden evaluations, thereby providing incremental theoretical and empirical value.

Accordingly, the present study develops and evaluates a preliminary measure of fertility social mentality among women of reproductive age in China, with the aim of providing a conceptually clearer and psychometrically grounded tool for subsequent research.

## 2 Methods

### 2.1 Participants

A random sampling method was employed to conduct a survey among female populations aged 20–49 in regions such as Chongqing, Sichuan, and Hunan. The selection of this age range was based on the United Nations’ definition of women of reproductive age (15–49) (Kanem, 2018), combined with the legal marriage age in China (20 years old), and was consistent with the standards of previous studies (Qiu and Li, 2024). The sample size was calculated as 5–10 times the number of items (Tinsley and Tinsley, 1987). Questionnaires were deleted according to the following criteria: (1) the time taken to complete the questionnaire was too short; (2) the unanswered portion exceeded one-third; and (3) the responses showed a regular pattern.

A total of four batches of samples were collected in this study. Sample 1 was used for item analysis and exploratory factor analysis. Questionnaires were distributed via the Wenjuanxing online platform, and a total of 526 questionnaires were retrieved. After screening, 496 valid questionnaires were collected, with an effective response rate of 94.30%. Eighty-six individuals were randomly selected from the formal test sample for retesting after a four-week interval. Sample 2 was used for confirmatory factor analysis and reliability and validity analysis. Questionnaires were distributed both through the Wenjuanxing online platform and offline, and a total of 921 questionnaires were retrieved. After screening, 857 valid questionnaires were collected, with an effective response rate of 93.05%. Sample 3 was used for measurement invariance testing. Questionnaires were distributed via the Wenjuanxing online platform and offline, and a total of 688 questionnaires were retrieved. After screening, 607 valid questionnaires were collected, with an effective response rate of 88.23%. Sample 4 was used for criterion validity testing. Through the Wenjuanxing online platform and offline distribution, a total of 680 questionnaires were collected. After screening and excluding 48 questionnaires, 632 valid responses remained, yielding a valid response rate of 91.6%. Sample 5 was used to test the criterion validity of indicators related to fertility behavior. A total of 387 questionnaires were collected through the “Wenjuanxing” online platform and offline distribution. After screening and excluding 25 questionnaires, 362 valid responses remained, resulting in a response rate of 93.5%. Table 1 lists the basic information about the samples.

**Table 1:** Demographic information of the samples.

Sample	Variable	Group	*N*	Percentage
Sample 1	Age	20–29 years	163	32.9
		30–39 years	169	34.1
		40–49 years	164	33.1
	Marital Status	Unmarried	124	25.0
		Married	249	50.2
		Divorced	123	24.8
Sample 2	Age	20–29 years	528	61.6
		30–39 years	224	26.1
		40–49 years	105	12.3
	Marital status	Unmarried	305	35.6
		Married	431	50.3
		Divorced	121	14.1
Sample 3	Age	20–29 years	528	61.6
		30–39 years	224	26.1
		40–49 years	105	12.3
	Marital status	Unmarried	143	23.6
		Married	328	54.0
		divorced	136	22.4
Sample 4	Age	20–29 years	426	67.4
		30–39 years	167	26.4
		40–49 years	39	6.2
	Marital status	Unmarried	498	78.8
		Married	131	20.7
		Divorced	3	0.5
Sample 5	Age	20–29 years	147	40.6
		30–39 years	183	50.6
		40–49 years	32	8.8
	Marital status	Unmarried	117	32.3
		Married	226	62.4
		Divorced	19	5.2

### 2.2 Scale development procedure

During this research phase, according to the deductive approach proposed by Hinkin (1998), the researchers systematically reviewed the literature and related studies on social mentality and the fertility psychology of women and constructed an initial item pool based on the research theme. In the process of constructing the item pool, scale items were divided into three categories: fertility social value, fertility social cognition, and fertility social emotion. These subdimensions draw on the established tool paradigm of social mentality, namely the framework of the social mentality index system constructed by Yang (2006). All initial items strictly reflected the fertility psychological characteristics of women of childbearing age and were presented in a concise and straightforward manner to avoid overlapping content.

Second, content validity was examined. This study invited eight experts in psychology and sociology, including five professors and three associate professors whose research areas covered social psychology, demography, and women’s studies, to form an expert panel. The panel rated all items on a 4-point scale ranging from 1 (not relevant) to 4 (very relevant) based on item relevance to the construct being measured. Polit and Beck (2006) indicated that when the number of experts is ≤5, the item-level content validity index (I-CVI) should be 1.00, and when the number of experts is ≥6, the I-CVI should be ≥0.78. The results showed that I-CVI values ranged from 0.88 to 1.00, all exceeding the acceptable standard of 0.78. The scale-level content validity index (S-CVI) was 0.96, indicating that the initial scale demonstrated good content validity (Polit and Beck, 2006).

Finally, a preliminary questionnaire consisting of 38 items across three dimensions was formed. A 5-point Likert scale was used, with scores from 1 to 5 corresponding to very inconsistent to very consistent. Ten items (6, 8, 15, 18, 23, 25, 26, 27, 28, and 37) were reverse scored. Higher scores indicated a more positive fertility social mentality. Respondents were required to answer based on their actual situation.

### 2.3 Measure

#### 2.3.1 Fertility Efficacy Scale

This study used the Fertility Efficacy Scale as the criterion measure. This scale was adapted by Chen et al. (2024) from the General Self-Efficacy Scale (GSE). Typical items include “As long as I put in the effort, I can resolve most problems encountered during the process of having children” and “I believe I am capable of handling the task of raising children.” The scoring range is “1 = Strongly Disagree; 4 = Strongly Agree.” Cronbach’s *α* for this scale is 0.91. In this study, its Cronbach’s α was 0.93.

#### 2.3.2 Fertility behavior scale

The measurement of fertility behavior draws on the methodology of Miller (1986) and employs the version adapted for the Chinese context by Li et al. (2025). The measure includes one item: “Have you tried to get pregnant?” It uses a 6-point scale, where scores 0–5 correspond to: “No, I am using contraception,” “No, I sometimes use contraception and sometimes do not,” “No, I am not using contraception,” “Yes, I am passively trying to conceive,” “Yes, I sometimes actively try to conceive and sometimes passively try to conceive,” and “Yes, I am actively trying to conceive,” respectively assigned scores of 0–5. Higher scores indicate stronger fertility behavior.

### 2.4 Data analyses

SPSS 27.0 was used to conduct item analysis, exploratory factor analysis (EFA), reliability analysis, and correlation analysis. For item analysis, the critical ratio method and the item–total correlation method were applied. First, total questionnaire scores were calculated and ranked. In accordance with conventional psychometric standards (Wu, 2010), participants in the top and bottom 27% were classified as high- and low-scoring groups, respectively. Independent samples t-tests were conducted to examine differences in item scores between the two groups. Pearson correlation coefficients were then calculated between each item score and the total score. Items were deleted if correlations were not statistically significant (*p* > 0.05) or if coefficients were below the critical value of 0.40.

For EFA, data suitability was first evaluated using the Kaiser–Meyer–Olkin (KMO) measure and Bartlett’s test of sphericity. Factors were extracted using principal axis factoring with varimax rotation. Items were screened and eliminated based on the following criteria (Carpenter, 2018; Peterson, 2000): (1) communality <0.40; (2) factor loading <0.50; (3) cross-loading; (4) misclassified or theoretically uninterpretable items; and (5) factors containing fewer than three items.

Subsequently, confirmatory factor analysis (CFA) and measurement invariance tests were conducted using Mplus 8.3. Three competing models were specified and evaluated via CFA. Measurement invariance was tested across two grouping variables: age and marital status. First, a configural invariance model (M1) was specified to test the equivalence of the basic factor structure. Second, assuming M1 was supported, metric invariance (weak invariance) model M2 was tested by comparing its fit with M1. Third, assuming M2 was supported, scalar invariance (strong invariance) model M3 was tested by comparing its fit with M2. Changes in model fit indices were evaluated using the criteria ΔCFI ≤ 0.01, ΔTLI ≤ 0.01, and ΔRMSEA ≤ 0.015 (Vandenberg and Lance, 2000).

## 3 Results

### 3.1 Item analysis

Independent samples *t*-tests were conducted using Sample 1 (*N* = 496). Results showed that all item scores differed significantly between the extreme groups (*t* = 9.652–23.259, *p* < 0.001), and no items were deleted. Correlation analysis further indicated that item–total correlation coefficients ranged from 0.621 to 0.766, all exceeding the critical value of 0.40 (*p* < 0.001). Therefore, no items were removed at this stage.

### 3.2 Equations exploratory factor analysis

EFA was conducted on Sample 1 data (*N* = 496). The KMO value was 0.982, well above the recommended threshold of 0.800. Bartlett’s test of sphericity was significant (*χ*^2^ = 12777.530, *df* = 703, *p* < 0.001), indicating that the data met the requirements for factor analysis. Three factors with eigenvalues greater than 1 were extracted after rotation, explaining a cumulative variance of 59.437%. This supported the proposed three-factor structure. Through multiple rounds of EFA, items 15, 3, 24, 29, 11, 19, 12, 36, 13, 21, and 23 were deleted, resulting in 27 retained items. The final three-factor solution explained 62.200% of the cumulative variance, meeting psychometric standards (Table 2).

**Table 2:** Factor structure from EFA of the Fertility Social Mentality Scale for Women of Childbearing Age.

Items	F1	F2	F3	Communality
1. Having children can create a lively family atmosphere	0.662			0.582
4. Children are the bond of family relationships	0.695			0.612
9. The arrival of a child brings joy to the family	0.663			0.537
14. Children are an emotional anchor	0.690			0.603
31. Children are the continuation of life	0.685			0.545
32. I enjoy the role of being a mother	0.531			0.543
34. I enjoy the process of accompanying my child’s growth	0.698			0.619
35. I like children	0.692			0.572
28. Having children is an affirmation and respect for life	0.651			0.637
30. Having children is an experience of happiness in life	0.593			0.554
16. Children are the fruit of a couple’s love	0.543			0.497
10. Having children can bring economic returns		0.623		0.568
2. The more children, the more prosperous the family		0.635		0.565
5. Having children can continue the family line		0.573		0.567
17. Not having children is a sign of being unfilial		0.664		0.573
33. I believe maternity leave basically meets the needs		0.554		0.524
22. I am very satisfied with the current societal environment for fertility		0.625		0.614
20. I believe current educational resources are fair		0.673		0.635
7. I believe current fertility medical resources are balanced		0.639		0.605
25. Having children will increase my physical burden			0.612	0.523
26. Having children will increase my psychological burden			0.643	0.602
27. Having children will limit my career development			0.653	0.606
18. I worry that having children will cause my figure to change for the worse			0.588	0.595
8. I worry that having children will lower my original quality of life			0.574	0.575
37. I worry that the supporting childcare facilities are inadequate			0.515	0.533
6. I am uncertain about having the ability to have children			0.589	0.562
38. I have no confidence in the future development of the societal fertility rate			0.584	0.578
Eigenvalues	13.663	1.876	1.255	
Variance contribution rate	22.908	19.302	15.297	

Fertility social value = F1, fertility social cognition = F2, fertility social emotion = F3.

In summary, EFA identified a three-factor structure comprising 27 items, forming the final Fertility Social Mentality Scale for Women of Childbearing Age. The scale includes three subdimensions: fertility social value (Factor 1, 11 items), fertility social cognition (Factor 2, 8 items), and fertility social emotion (Factor 3, 8 items).

### 3.3 Validity analysis

#### 3.3.1 Confirmatory factor analysis

**Figure 1:**
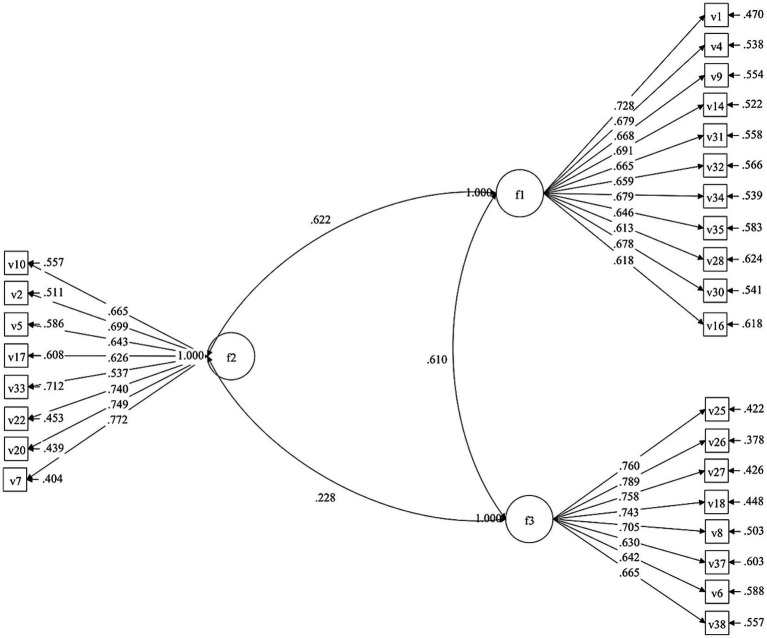
Standardized path diagram of fertility social mentality structure.

To further examine the scale structure, CFA was conducted using Sample 2 (*N* = 857). As shown in Table 3, Figure 1, the three-factor model demonstrated a better fit than the two-factor and one-factor models. Fit indices were *χ*^2^/*df* = 3.735, CFI = 0.918, TLI = 0.910, SRMR = 0.065, and RMSEA = 0.056 (90% CI: 0.053–0.060). All indices met acceptable criteria (Wen et al., 2004), confirming that the three-factor model exhibited good structural validity.

**Table 3:** Model fit indices for the Fertility Social Mentality Scale for Women of Childbearing Age.

Model	*df*	*χ* ^2^	*χ*^2^/*df*	CFI	TLI	SRMR	RMSEA
Three-factor	321	1198.895	3.735	0.918	0.910	0.065	0.056
Two-factor	323	2379.603	7.368	0.808	0.791	0.092	0.086
One-factor	324	4204.893	12.978	0.637	0.607	0.118	0.118

Three-factor model: fertility social value, fertility social cognition, fertility social emotion; two-factor model: fertility social value + fertility social cognition, fertility social emotion; one-factor model: fertility social value + fertility social cognition + fertility social emotion.

#### 3.3.2 Convergent validity

Convergent validity analysis (Table 4) showed that the average variance extracted (AVE) values for the three dimensions were 0.444, 0.466, and 0.509. Thus, the scale demonstrated adequate convergent validity. In addition, composite reliability (CR) values were 0.796, 0.765, and 0.809, all exceeding the recommended threshold of 0.70. Although the ideal AVE exceeds 0.50 (Fornell and Larcker, 1981), the AVE values for the two dimensions in this study are slightly below this threshold; however, their CR values are all above 0.70. Overall, the scale’s convergent validity remains at an acceptable level.

**Table 4:** Convergent validity and composite reliability indices for each dimension.

Items	F1	F2	F3
AVE	0.444	0.466	0.509
CR	0.796	0.765	0.809

Fertility social value = F1, fertility social cognition = F2, fertility social emotion = F3.

#### 3.3.3 Discriminant validity

The results of the discriminant validity test (Table 5) showed that the correlation coefficients between variables were all lower than the square root of the AVE. This indicates that the scale has good discriminant validity.

**Table 5:** Discriminant validity results.

Dimension	F1	F2	F3
F1	1		
F2	0.622	1	
F3	0.610	0.228	1
Square root of the AVE	0.667	0.683	0.714

Fertility social value = F1, fertility social cognition = F2, fertility social emotion = F3.

#### 3.3.4 Criterion validity

The criterion validity results (Tables 6, 7) showed that the Fertility Efficacy Scale was significantly positively correlated with the total score of fertility social mentality (*r* = 0.727, *p* < 0.001) and its dimensions of fertility social value (*r* = 0.698, *p* < 0.001), fertility social cognition (*r* = 0.639, *p* < 0.001), and fertility social emotion (*r* = 0.468, *p* < 0.001). In addition, fertility behavior was significantly positively correlated with the total score of fertility social mentality (*r* = 0.551, *p* < 0.001), fertility social value (*r* = 0.403, *p* < 0.001), fertility social cognition (*r* = 0.450, *p* < 0.001), and fertility social emotion (*r* = 0.416, *p* < 0.001).

**Table 6:** Criterion validity results of fertility efficacy.

Variable	Overall scale	F1	F2	F3
Overall scale	1			
F1	0.875^***^	1		
F2	0.877^***^	0.711^***^	1	
F3	0.744^***^	0.438^***^	0.471^***^	1
Fertility efficacy	0.727^***^	0.698^***^	0.639^***^	0.468^***^

^***^p < 0.001.

Fertility social value = F1, fertility social cognition = F2, fertility social emotion = F3.

**Table 7:** Criterion validity results of fertility behavior.

Variable	Overall scale	F1	F2	F3
Overall scale	1			
F1	0.861^***^	1		
F2	0.775^***^	0.554^***^	1	
F3	0.596^***^	0.219^***^	0.261^***^	1
Fertility behavior	0.551^***^	0.403^***^	0.450^***^	0.416^***^

^***^p < 0.001.

Fertility social value = F1, fertility social cognition = F2, fertility social emotion = F3.

### 3.4 Reliability analysis

#### 3.4.1 Composite reliability

The CR analysis (Table 4) showed that the CR coefficients for each dimension of the Fertility Social Mentality Scale ranged from 0.765 to 0.809, all exceeding the critical value of 0.70. This result confirms that the items within each factor have a high degree of internal consistency.

#### 3.4.2 Internal consistency reliability

The results of the data analysis (Table 8) showed that the overall Cronbach’s *α* for the scale reached 0.924, and the alpha coefficient for each dimension remained above 0.70. These results indicate that the measurement tool exhibits good internal consistency across different dimensions and can stably reflect the social mentality characteristics of women of childbearing age.

**Table 8:** Internal consistency reliability results.

Coefficient	F1	F2	F3	Overall scale
Cronbach’s *α*	0.897	0.871	0.892	0.924

Fertility social value = F1, fertility social cognition = F2, fertility social emotion = F3.

#### 3.4.3 Test–retest reliability

After an interval of 4 weeks, a retest was conducted with 86 participants. The results (Table 9) showed that the test–retest reliability of the Fertility Social Mentality Scale for Women of Childbearing Age was 0.953, and the values for each dimension were 0.949, 0.924, and 0.890, respectively. This indicates that the scale has good stability.

**Table 9:** Test–retest reliability results.

Coefficient	F1	F2	F3	Overall scale
Test–retest reliability coefficient	0.949	0.924	0.890	0.953

Fertility social value = F1, fertility social cognition = F2, fertility social emotion = F3.

### 3.5 Measurement invariance

The analysis indicated that the data supported the configural invariance of the three-dimensional measurement structure of the Fertility Social Mentality Scale for Women of Childbearing Age across age and marital status, indicating that each model demonstrated good fit. Furthermore, metric invariance and scalar invariance were also supported, as changes in model fit indices did not exceed the recommended critical values (Vandenberg and Lance, 2000). The results are presented in Tables 10, 11.

**Table 10:** Results of measurement invariance testing across age groups.

Model	Invariance model	*χ* ^2^	*df*	RMSEA	CFI	TLI	SRMR	ΔCFI	ΔTLI	ΔRMSEA
M1	Configural invariance	2334.289	972	0.083	0.874	0.863	0.061	——	——	——
M2	Metric invariance (weak invariance)	2391.766	1,024	0.081	0.873	0.870	0.060	0.001	0.007	0.002
M3	Scalar invariance (strong invariance)	2480.537	1,076	0.080	0.870	0.873	0.072	0.003	0.003	0.001

**Table 11:** Results of measurement invariance testing across marital status.

Model	Invariance model	*χ* ^2^	*df*	RMSEA	CFI	TLI	SRMR	ΔCFI	ΔTLI	ΔRMSEA
M1	Configural invariance	2287.036	972	0.082	0.878	0.868	0.060	——	——	——
M2	Metric invariance (weak invariance)	2402.228	1,024	0.082	0.872	0.868	0.078	0.006	<0.001	<0.001
M3	Scalar invariance (strong invariance)	2499.052	1,076	0.081	0.868	0.870	0.082	0.004	0.002	0.001

## 4 Discussion

Taken together, the findings indicate that the Fertility Social Mentality Scale for Women of Childbearing Age has promising internal psychometric characteristics, including a coherent factor structure, satisfactory internal consistency, acceptable temporal stability, and measurement invariance across age and marital status. On this basis, the scale may be used as a preliminary tool for assessing fertility social mentality among women of childbearing age in China.

### 4.1 The structure of social mentality toward childbearing among women of childbearing age

This study employed methods including item analysis, EFA, and CFA to develop the Fertility Social Mentality Scale for Women of Childbearing Age and to determine its three-dimensional structure: fertility social value, fertility social cognition, and fertility social emotion. This structure aligns with the basic delineation of cognition, affect, and value dimensions in social mentality theory (Yang, 2006) while also reflecting the specific characteristics of fertility behavior and its unique socio-cultural implications. Moreover, during dimension construction, “Social Behavioral Intention” was not established as a separate dimension, based on three academic considerations. First, emotion, cognition, and value form a psychological continuum that captures the core psychological logic of fertility social mentality, whereas behavioral intention, as a precursor to action, more strongly reflects the interaction between external structural factors and individual decision-making. Its inclusion could blur the boundary between psychological and external drivers, potentially reducing measurement reliability and validity. Second, fertility decision-making is a composite outcome of interactions among willingness, negotiation, and norms; behavioral intention inherently integrates multidimensional evaluations such as fertility costs, emotional value, and trust in policies. Establishing it as an independent dimension could result in conceptual overlap with the value orientation and social cognition dimensions. Third, fertility social mentality emphasizes diachronic dynamic evolution and requires capturing real-time fluctuations in emotion, cognition, and value, whereas behavioral intention reflects synchronic decision-making tendencies. Therefore, the three-dimensional structure adopted in this study more effectively captures the core connotations and dynamic characteristics of fertility social mentality among women of childbearing age. The specific connotations and structure of each dimension are described below.

The Fertility Social Value dimension consists of 11 items and primarily reflects individuals’ evaluative standards for fertility behavior and their criteria for selecting desired goals among multiple alternatives. This dimension includes family emotional value, internal preference, responsibility orientation, participation judgment, and investment preparedness. Family emotional value reflects the importance of fertility for family continuity and emotional bonding. For example, many women of childbearing age view fertility as an essential component of family happiness and completeness (Ding and Zhao, 2024; Xu and Feng, 2025). Responsibility orientation emphasizes fertility as a form of social responsibility or family obligation, such as the belief that childbearing fulfills the responsibility of continuing the family lineage (Xu and Guo, 2024). Participation judgment and investment preparedness reflect individuals’ initiative and planning in fertility decision-making, including consideration of factors such as family support prior to childbearing (Minello and Russo, 2025; Sun et al., 2025). In summary, this dimension aligns with existing research on the intergenerational transmission of fertility values (Murphy, 2012) and the evolution of traditional notions such as “the more children, the better” in modern society (Zheng, 2025), indicating that societal values regarding fertility are both embedded in culture and moderated by individuals’ rational assessments. This dimension reveals the multiple value orientations underlying fertility behavior and provides an important basis for understanding the complexity of fertility decision-making among women of childbearing age.

The Fertility Social Cognition dimension consists of 8 items and primarily reflects individuals’ understanding of fertility behavior and related social norms. This dimension includes traditional cultural cognition, happiness and growth cognition, policy support cognition, and family support cognition. Traditional cultural cognition reflects recognition of the importance of fertility for family continuity and lineage. For example, many women regard fertility as an embodiment of the traditional belief that “more children bring more happiness” (Wei et al., 2024). Happiness and growth cognition captures awareness of the emotional fulfillment and personal growth associated with childbearing, such as the belief that raising children brings joy through accompanying their development (Qian and Huang, 2024). Policy support cognition and family support cognition reflect perceptions of the role of social policies and family assistance in fertility decisions. For example, many women believe that comprehensive social support policies can significantly alleviate fertility-related pressures (Li et al., 2025). This dimension highlights the multilayered cognitive processes underlying fertility behavior and offers guidance for developing targeted fertility support policies. Furthermore, this supports the feasibility of applying social cognitive theory (Bandura, 2002) to fertility research, namely that individuals’ expectations regarding fertility and their understanding of social norms are jointly shaped by direct experience, vicarious experience, and social persuasion within the interplay of individual, social, and collective agency.

The Fertility Social Emotion dimension consists of 8 items and reflects individuals’ emotional responses to fertility behavior. It includes risk and crisis perception, satisfaction, and social trust and confidence. Risk and crisis perception captures concerns about potential economic, career, and health risks associated with childbearing, such as worries about career disruption (Yang and Lu, 2025). Satisfaction reflects subjective emotional experiences during the fertility process, including evaluations of the balance between costs and rewards of having children (Fredrickson, 2001). Social trust and confidence represent trust in fertility-related social support systems and confidence in achieving fertility goals. For example, many women believe that societal support and family care enhance confidence in childbearing decisions (Minello and Russo, 2025). This dimension provides insight into emotional experiences during the fertility process and offers a basis for improving fertility satisfaction and social trust among women of childbearing age. Additionally, it contrasts with negative emotions such as “fertility anxiety” and “parenting concerns” (Zhang and Zhao, 2022) as well as positive emotions such as “fertility well-being” and “sense of maternal accomplishment” (Kohler and Mencarini, 2016) found in existing research, indicating that social emotions regarding childbearing constitute a two-dimensional structure encompassing both risk perception and positive experiences. This finding also enriches the operationalization of emotional components within social mindset theory (Yang, 2006), providing a clear target for fostering a positive social mindset toward childbearing at the emotional level, which may in turn contribute to increasing fertility rates.

### 4.2 Reliability, validity and measurement invariance of the scale

This study examined the reliability and validity of the Fertility Social Mentality Scale for Women of Childbearing Age using multiple methods. The results indicated that the scale has sound psychometric properties. In addition, measurement invariance across age and marital status was tested.

The CR values ranged from 0.765 to 0.809, indicating good internal consistency. The overall Cronbach’s *α* was 0.924, and the alpha values for each subdimension exceeded 0.70. Test–retest reliability ranged from 0.890 to 0.953. These results suggest that the scale demonstrates high stability and consistency (Wu, 2010).

CFA results showed that all fit indices reached acceptable levels: χ^2^/*df* = 3.735, CFI = 0.918, TLI = 0.910, SRMR = 0.065, and RMSEA = 0.056 (90% CI: 0.053–0.060). These values meet established criteria of RMSEA < 0.08, CFI and TLI > 0.90, SRMR < 0.08, and χ^2^/*df* < 5 (Schreiber et al., 2006). Convergent and discriminant validity analyses further indicated that the three dimensions of the scale have good distinctiveness while remaining moderately interrelated, which is consistent with theoretical expectations. It is worth noting that in this study, the AVE values for two dimensions were slightly below the 0.5 threshold; however, Rubia (2019) argues that the criterion of AVE > 0.50 may be difficult to meet and may obscure the assessment of actual convergent validity. He suggests that more flexible criteria should be adopted, taking into account the number of items, factor loadings, and reliability; provided these indicators meet the standards, an AVE value of less than 0.50 may still reflect an acceptable level of convergent validity. Furthermore, previous studies have reported similar findings, namely that while the AVE is slightly below 0.50, the overall psychometric properties remain acceptable (Qin et al., 2024; Jiang et al., 2025; Dadandı and Aydın, 2025; Ren et al., 2026). Additionally, the criterion validity analysis revealed that both the total score and the scores on each dimension of the Fertility Social Mentality Scale were significantly positively correlated with the scores on the Fertility Efficacy Scale, indicating that the scale possesses good criterion validity. Individuals with a higher level of fertility social mentality also exhibit stronger coping beliefs regarding childbearing-related tasks and challenges. On the one hand, positive social cognitions regarding childbearing help individuals rationally assess the difficulties and resources involved in the childbearing process, thereby enhancing their fertility efficacy (Xu et al., 2026). On the other hand, stable childbearing value orientations provide individuals with intrinsic motivation, encouraging them to believe in their ability to accomplish childbearing-related tasks (Tartakovsky and Mizrahi, 2025). At the same time, positive emotional experiences related to childbearing may indirectly enhance individuals’ positive evaluations of their own reproductive capabilities by reducing the depletion of psychological resources (Mijalevich-Soker et al., 2025). Similarly, the results of the criterion validity analysis show that fertility behavior is significantly and positively correlated with both the total score and all dimensions of the fertility social mentality, indicating that the Fertility Social Mentality Scale has good concurrent criterion validity in relation to individuals’ actual fertility behavior. Positive fertility social cognitions, positive emotional experiences related to childbearing, and supportive childbearing values collectively contribute to the formation of a positive fertility social mentality, thereby increasing the likelihood of actual childbearing behavior; conversely, a negative fertility social mentality may act as a psychological barrier to fertility behavior. In summary, the fertility social mentality is not only significantly associated with fertility efficacy but also demonstrates concurrent validity with actual fertility behavior.

Measurement invariance testing showed that configural, metric, and scalar invariance models were all supported. These results indicate that the scale demonstrates stable measurement properties across age and marital status. Thus, measurement invariance of fertility social mentality among women of childbearing age across different subgroups was fully established. This evidence supports the cross-group comparability of the scale and its ability to capture both shared and subgroup-specific characteristics of fertility social mentality.

### 4.3 Limitations and future research

This study still has several limitations. First, the scale was developed using cross-sectional data; future studies should incorporate longitudinal designs to further examine the stability of its measurement structure. Second, in determining the initial items for the scale, this study drew upon the deductive approach proposed by Hinkin (1998). The initial item pool was primarily constructed based on a systematic literature review, and no qualitative interviews or focus group discussions were conducted with women of childbearing age; this may have limited the items’ ability to reflect the target group’s actual experiences and perspectives. Therefore, future research might incorporate qualitative research methods to further validate and enrich the existing items. Third, It is worth noting that while the relatively high composite reliability (CR) confirms the scale’s good internal consistency, the average variance extracted (AVE) values for some dimensions, which are slightly below 0.50, suggest that there is still room for future research to optimize the measurement indicators, thereby further increasing the variance explained by the relevant dimensions. Fourth, fertility is a global issue, yet the sample consisted primarily of women from China, which may limit the generalizability of the scale. Fertility social mentality may vary across regions due to differences in geography, economic development, and cultural practices. Future research should refine the scale and expand sampling to broader socio-cultural contexts to enhance its validity and applicability. Fifth, as social conditions, fertility policies, and social structures continue to evolve, the factors influencing fertility social mentality are also changing. Future research should account for these dynamic influences, update scale content as needed, and ensure that the instrument accurately reflects current conditions and developmental trends over time. This will provide a standardized, timely, and forward-looking measurement tool for related research. Finally, the present operationalization of fertility social mentality encompasses cognitive, emotional, and value-based components, which bears structural resemblance to general attitudinal frameworks. Although these dimensions were contextualized within the specific domain of fertility, we acknowledge that the distinctiveness of the construct from existing fertility-related attitudinal measures has not been empirically established in the current study. Future research should employ incremental validity designs, such as hierarchical regression or structural equation modeling, to examine whether fertility social mentality accounts for variance in relevant outcomes (e.g., fertility intentions, fertility decision-making) beyond established fertility-related measures.

## 5 Conclusion

The Fertility Social Mentality Scale for Women of Childbearing Age consists of 27 items across three dimensions: fertility social value, fertility social cognition, and fertility social emotion. The scale demonstrates good reliability, validity, and measurement invariance. Its broader applicability and practical implications, however, should be further examined in future studies using external validation criteria.

## Data Availability

The raw data supporting the conclusions of this article will be made available by the authors, without undue reservation.
