# Supplementary material for: Fertility Social Mentality Scale for Women of Childbearing Age: a scale development study
Source: Front Psychol. 2026 Jun 11;17:1799068. doi: 10.3389/fpsyg.2026.1799068 (PMC13293805; doi:10.3389/fpsyg.2026.1799068)
Supplement: Supplementary file 1 [file Supplementary_File_1.docx]

Supplementary Material

# Fertility Social Mentality Scale for Women of Childbearing Age

| Items | Very Inconsistent | Inconsistent | Neutral | Consistent | Very Consistent |
| --- | --- | --- | --- | --- | --- |
| 1. Having children can create a lively family atmosphere | 1 | 2 | 3 | 4 | 5 |
| 2. The more children, the more prosperous the family | 1 | 2 | 3 | 4 | 5 |
| 3. Children are the bond of family relationships | 1 | 2 | 3 | 4 | 5 |
| 4. Having children can continue the family line | 1 | 2 | 3 | 4 | 5 |
| 5. I am uncertain about having the ability to have children | 1 | 2 | 3 | 4 | 5 |
| 6. I believe current fertility medical resources are balanced | 1 | 2 | 3 | 4 | 5 |
| 7. I worry that having children will lower my original quality of life | 1 | 2 | 3 | 4 | 5 |
| 8. The arrival of a child brings joy to the family | 1 | 2 | 3 | 4 | 5 |
| 9. Having children can bring economic returns | 1 | 2 | 3 | 4 | 5 |
| 10. Children are an emotional anchor | 1 | 2 | 3 | 4 | 5 |
| 11. Children are the fruit of a couple's love | 1 | 2 | 3 | 4 | 5 |
| 12. Not having children is a sign of being unfilial | 1 | 2 | 3 | 4 | 5 |
| 13. I worry that having children will cause my figure to change for the worse | 1 | 2 | 3 | 4 | 5 |
| 14. I believe current educational resources are fair | 1 | 2 | 3 | 4 | 5 |
| 15. I am very satisfied with the current societal environment for fertility | 1 | 2 | 3 | 4 | 5 |
| 16. Having children will increase my physical burden | 1 | 2 | 3 | 4 | 5 |
| 17. . Having children will increase my psychological burden | 1 | 2 | 3 | 4 | 5 |
| 18. Having children will limit my career development | 1 | 2 | 3 | 4 | 5 |
| 19. Having children is an affirmation and respect for life | 1 | 2 | 3 | 4 | 5 |
| 20. Having children is an experience of happiness in life | 1 | 2 | 3 | 4 | 5 |
| 21. Children are the continuation of life | 1 | 2 | 3 | 4 | 5 |
| 22. I enjoy the role of being a mother | 1 | 2 | 3 | 4 | 5 |
| 23. I believe maternity leave basically meets the needs | 1 | 2 | 3 | 4 | 5 |
| 24. I enjoy the process of accompanying my child's growth | 1 | 2 | 3 | 4 | 5 |
| 25. I like children | 1 | 2 | 3 | 4 | 5 |
| 26. I worry that the supporting childcare facilities are inadequate | 1 | 2 | 3 | 4 | 5 |
| 27. I have no confidence in the future development of the societal fertility rate | 1 | 2 | 3 | 4 | 5 |

Note: Items 5, 7, 13, 16, 17, 18, 26, and 27 are reverse-scored.
